# International regulatory agencies: Indian clinical trials

**DOI:** 10.4103/2229-3485.76290

**Published:** 2011

**Authors:** Arun Bhatt

**Affiliations:** *Clininvent Research Pvt. Ltd., A-103, Everest Chambers, Marol Naka, Andheri - Kurla Road, Andheri (E), Mumbai 400 059, India. E-mail: arunbhatt@clininvent.com*

**Table:** Number of patients in pivotal trials submitted in MAAs to the EMA 2005–2009

Region	Number of patients	%	Number of sites	%	Number of patients per site
EU/EEA/EFTA	231,369	38.8	16,063	36.5	14
North America	209,750	35.2	19,362	44.0	11
Central/South America	54,657	9.2	2,625	6.0	21
Middle East/Asia/Pacific	46,505	7.9	2,577	5.9	18
CIS region	22,664	3.8	1,566	3.6	14
Africa	17,915	3	707	1.6	25
Australia–New Zealand	8,678	1.5	919	2.1	9
Eastern Europe–non EU	4,042	0.7	215	0.5	19
Total	595,580		44,034		

**Table:** 2005–2009

	No of CTs	No of sites	No of patients	No of patients per trial
China	17	185	4,417	260
India	65	528	8,920	137
Korea	58	291	3,320	57

Source: European Medicines Agency (EMA) clinical trials submitted in marketing authorization applications to the EMA overview of patient recruitment and the geographical location of investigator sites, 5 November 2010, EMA/INS/GCP/154352/2010 Compliance and Inspection

**Table:** Subjects and sites by country for marketing applications approved in the fiscal year 2008

Country	Number of patients	% of total patients	Number of sites	% of total sites	Number of patients per site
China	424	0.5	32	0.3	13
India	384	0.4	49	0.4	8
Korea	409	0.4	32	0.3	13
USA	40,039	43.1	5,098	45.4	8
All countries	92,859		11,227		

Source: Challenges to FDA’s ability to monitor and inspect foreign clinical trials, DHSS, June 2010

